# Design and Implementation of an EEG-Based Learning-Style Recognition Mechanism

**DOI:** 10.3390/brainsci11050613

**Published:** 2021-05-11

**Authors:** Bingxue Zhang, Chengliang Chai, Zhong Yin, Yang Shi

**Affiliations:** Department of Optical-Electrical & Computer Engineering, University of Shanghai for Science and Technology, Shanghai 200093, China; zhangbingxue@usst.edu.cn (B.Z.); andrewtsa11@st.usst.edu.cn (C.C.); 193780684@st.usst.edu.cn (Y.S.)

**Keywords:** learning-style recognition, EEG features, brain-computer interface, processing dimension, Felder–Silverman learning-style

## Abstract

Existing methods for learning-style recognition are highly subjective and difficult to implement. Therefore, the present study aimed to develop a learning-style recognition mechanism based on EEG features. The process for the mechanism included labeling learners’ actual learning styles, designing a method to effectively stimulate different learners’ internal state differences regarding learning styles, designing the data-collection method, designing the preprocessing procedure, and constructing the recognition model. In this way, we designed and verified an experimental method that can effectively stimulate learning-style differences in the information-processing dimension. In addition, we verified the effectiveness of using EEG signals to recognize learning style. The recognition accuracy of the learning-style processing dimension was 71.2%. This result is highly significant for the further exploration of using EEG signals for effective learning-style recognition.

## 1. Introduction

### 1.1. Overview of Learning Styles

Learning style is a relatively stable learning preference that learners gradually form in long-term learning activities [1]. It is an important factor reflecting learners’ individual differences [2]. Personalized learning strategies, content, and resources can be formulated by analyzing and studying different learning styles, which can improve learning efficiency and enthusiasm [3]. Therefore, accurately recognizing learners’ learning styles not only is necessary for personalized teaching but also has important research significance and application value for implementing modern education modes [4].

**Figure 1:**
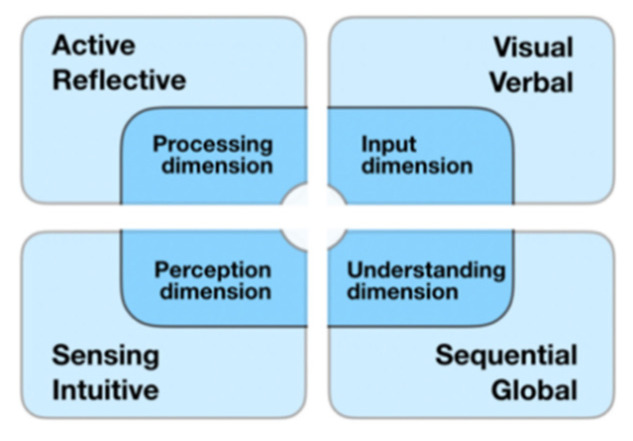
Felder–Silverman learning-style model.

The earliest theory of learning style was proposed by Thelen in 1954 [5]. After that, researchers put forward various learning-style models [6], including Kolb [7], Felder–Silverman [8], VARK [9], and Gregorc [10]. Among them, the Felder–Silverman model has been widely recognized and adopted for well-known adaptive learning systems such as CS383, MASPLANG, LSAS, and TANGOW. Supported by a large amount of experimental data, the Felder–Silverman model has been shown to have good applicability and effectiveness [11]. The Felder–Silverman model divides learning style into four dimensions: information processing, perception, input, and understanding. Each dimension has two types of learning styles, as shown in Figure 1.

### 1.2. Current State of Learning-Style Recognition Methods

At present, there are two main ways to recognize learning style: explicit recognition and implicit recognition.

(1) Explicit recognition calculates scores from the Index of Learning Styles (ILS) questionnaire [12] to judge subjects’ learning styles [13]. Researchers such as Surjono [14], Hwang [15], and Wang [16] have built learning-style models based on ILS. Table 1 summarizes the advantages and disadvantages of explicit recognition.
(2) Implicit recognition mines and analyzes learners’ interactive behavior data using online learning systems (e.g., learning behavior logs and social behavior data) to indirectly grasp learning styles. Thus, there is no need for participants to fill out the ILS. Many researchers have studied the implicit recognition mechanism. Taking the number of clicks on certain buttons, time spent on activities, quiz results, number of posts in forums, and other behavior data as inputs, Cha et al. [17] used a decision tree and hidden Markov model to recognize learning styles. Villaverde et al. [18], meanwhile, used the following as input sources: which types of learning materials learners prefer, whether learners modified answers before submitting, and whether learners actively participated in forums; on that basis, artificial neural networks were used for recognition. Subsequent studies that used online interactive behavior for learning-style recognition have employed decision trees [19,20], Bayesian networks [21], neural networks [22,23], genetic algorithms [24], and the J48 algorithm [25]. The abovementioned studies all used conventional online learning-behavior features as their data sources; our study, however, used EEG signals for learning-style recognition, the advantages of which will be discussed below.

Table 1 summarizes the advantages and disadvantages of implicit recognition methods.

**Table 1:** Advantages and disadvantages of current learning-style recognition methods.

Method	Advantages	Disadvantages
Explicit recognition	(1) ILS is customized according to the learning-style model. The reliability and validity of ILS have theoretical support, showing high authority [26].	(1) It is hard for learners to understand the concepts of learning styles; thus, they might not be able to accurately fill out the questionnaire [26]. (2) When learners respond to the ILS, they will have a subjective bias toward the test results, thus affecting the objectivity of the results [26]. (3) Calculating learning style based on a one-time questionnaire cannot reflect changes in characteristics over time [26].
Implicit recognition	(1) Recognition results are automatically obtained and classified by the system. Learners do not need additional time to fill out the questionnaire [26]. (2) Compared to explicit recognition, it is less affected by the learner’s subjective factors, and the data source is more objective [26].	(1) Given the problem of a “cold start,” it is necessary to obtain a large amount of learners’ online learning-behavior data for more accurate recognition [27]. (2) The credibility of the data source itself will have a great impact on the recognition results [28].

### 1.3. Applying EEG Signals to Learning-Style Recognition

Differences in the learning-style dimensions (i.e., information processing, perception, input, and understanding) reflect differences in the way learners analyze and solve problems [29]. This process is related to how the brain internalizes and understands information. It is difficult, therefore, to efficiently analyze subjects’ learning styles using the abovementioned conventional recognition methods. An electroencephalogram (EEG) is a record of spontaneous and rhythmic electrophysiological activity in the brain [30]. Its various bands can reflect the internal activity state of the brain (Table 2). EEGs have been widely used in emotion recognition [31,32], attention level measurement [33], cognitive workload measurement [34,35], thinking-state detection [36,37], academic stress detection [38], cognitive psychological disease detection [39,40], fatigue monitoring [41], mind control [42], and other areas. Since the biological nature of EEG information is difficult to disguise or mask, EEGs can more objectively reflect internal processes than behaviors, voices, facial expressions, and so on [43]. Therefore, applying EEGs to learning-style recognition has considerable potential. 

Among Felder–Silverman’s four dimensions, the processing dimension concerns how received information is processed into knowledge according to thinking processes [8]. Reflective learners are better at introspection and pay attention to their internal situation; active learners tend to practice and put their energy into the external environment [50]. The processing dimension can reflect differences in learners’ cognitive processing, which can have guiding significance for personalized teaching as well as lifelong learning. Therefore, this study used EEG features to analyze the processing dimension of learning style.

### 1.4. Experimental Questions

(1) How should an experimental method be designed to stimulate internal state differences in the processing dimension of learning styles?
(2) Can the student’s learning style be recognized based on EEG signals?

## 2. Experimental Design

**Figure 2:**
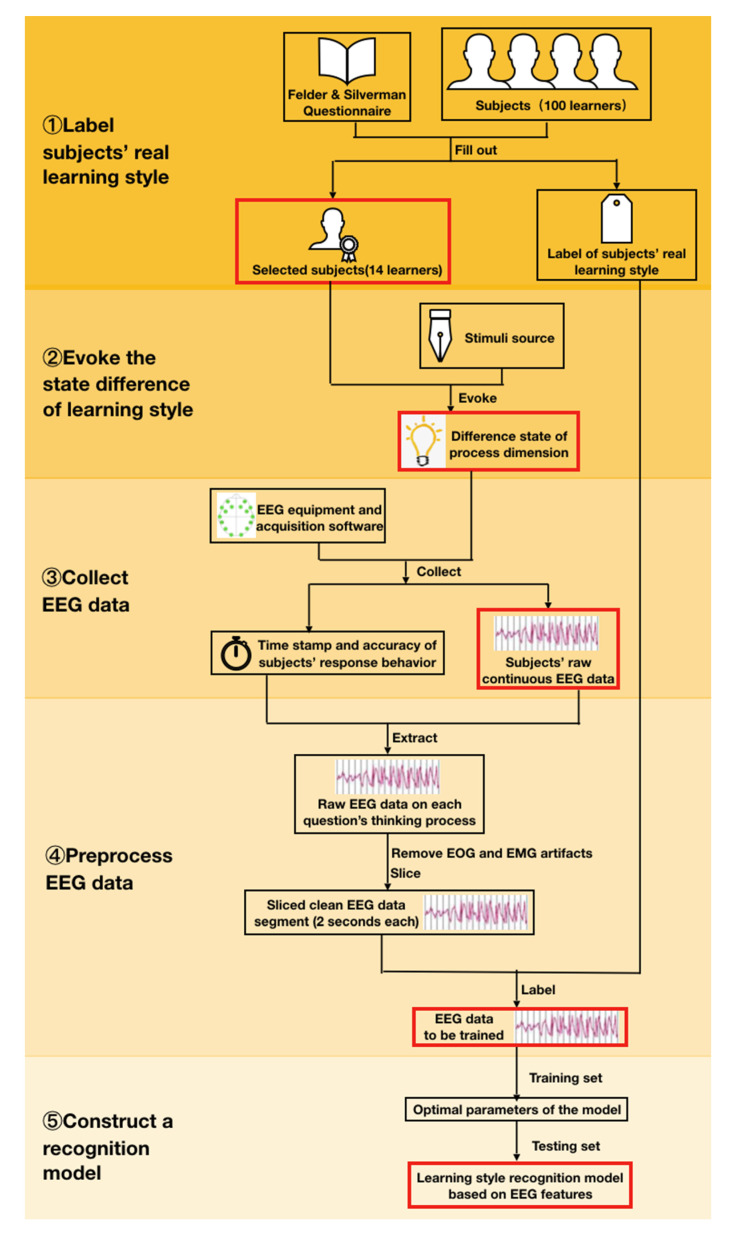
Experimental flowchart (red box is the output of each step).

The experimental procedure included five steps (Figure 2): 

(1) Label the subjects’ actual learning style. We recruited subjects to fill out the ILS and designed measures to ensure the accuracy of the results. We selected subjects who were willing to participate and who met the requirements for the follow-up experiment. (2) Evoke the subjects’ learning style-related internal state differences. The subjects’ different internal states in the processing dimension were stimulated through our designed stimuli. (3) Collect the EEG data. Methods were designed to collect EEG signals generated in the previous step to obtain the subjects’ raw EEG data. (4) Preprocess the EEG data. The raw EEG data were preprocessed and cleaned EEG for training. (5) Train the model to recognize learning style. Model training was performed using the data, and an EEG-based learning-style recognition model was finally constructed.

### 2.1. Labeling Subjects’ Real Learning Styles

The ILS was used to obtain and label the subjects’ actual learning styles. Then, we analyzed the distribution of learning styles. Based on the results and on the subjects’ willingness, we selected subjects for the follow-up experiments. The detailed steps are described below.

#### 2.1.1. Labeling Method

Felder and Soloman designed the ILS in 1997 to measure learning styles in the four dimensions of the Felder–Silverman model. The ILS has 11 questions for each dimension for a total of 44 questions. It has been widely used in research on learning-style recognition and has been shown to have good reliability and validity [11]. Therefore, we used the ILS to label the subjects’ learning styles. However, there are a couple of problems with using the ILS for this purpose. First, since people have different learning backgrounds, and the questions in the ILS are expressed in an abstract way, it might be difficult for subjects to fully understand the meaning of each question. Second, subjects might be reluctant to fill out the questionnaire and might answer randomly, which would produce large deviations in the results. 

In light of the above, we performed a straightforward, detailed translation of each ILS item and explained the meaning of each to the subjects verbally before they filled it out. Moreover, we asked the subjects about their willingness to fill out the ILS in advance, and only those who expressed willingness to do so were selected. They were asked to fill out the ILS based on careful consideration of their own actual situation. On this basis, subjects’ learning styles were obtained, providing a reliable basis for labeling learning styles.

#### 2.1.2. ILS Results

**Figure 3:**
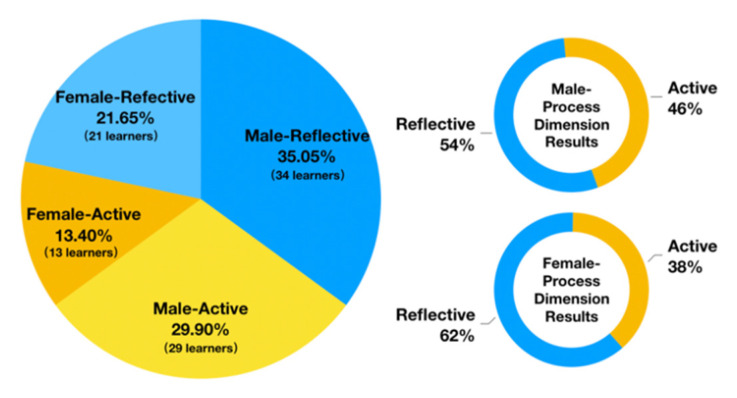
Distribution of learning styles among the subjects based on the ILS.

ILS questionnaires were distributed to 100 college students, and 97 were collected. The results were calculated to analyze the distribution of the subjects’ learning styles and to further screen the subjects for the experiments. According to the learning style results of all the 97 participants who completed the ILS in Figure 3, the Chi-square test [51] was conducted to verify the effect of gender on the processing dimension of learning style; we put forward the original hypothesis that “gender (male/female) has no significant effect on the processing dimension of learning style (reflective/active)”. The test result shows that χ^2^ statistic = 0. 2753 (degree of freedom=1), *p* = 0.5998, which cannot reject the original hypothesis. That is to say, there is not a statistically significant difference in gender proportions, so the variable of gender will no longer be considered in the subsequent experiments.

#### 2.1.3. Screening Subjects

To improve the quality of the experimental data, we needed to select qualified subjects. First, subjects whose ILS results were neutral—that is, the tendency to be reflective or active was not obvious—needed to be filtered. Second, to ensure balanced data distribution, the number of data sets for the two categories needed to be as close as possible. Third, the subjects needed to be willing to proceed to the next step. 

Based on the above, seven active and seven reflective subjects (each showing obvious active or reflective learning styles) were selected, for a total of 14 undergraduate subjects aged 18–21 (average: 19.4 years, standard deviation: 0.9 years). All subjects had normal hearing and vision and were right-handed. None were informed about the experimental hypotheses. Before the experiment, all subjects provided signed informed consent, indicating they fully understood the experimental procedure.

### 2.2. Evoking the State Difference of Learning Style

#### 2.2.1. Principles for Selecting the Stimulus Mode

Designing the stimuli was a key aspect of the experiment. Through the use of external stimuli, the desired internal state signals of the subjects would be generated, allowing us to analyze them. 

The processing dimension of the Felder–Silverman model concerns how individuals process information into knowledge. The behavioral differences between reflective and active learners are as follows: Reflective learners tend to think carefully about problems, take sufficient time to examine the problems, weigh various problem-solving methods, and then choose the best solution, thereby making fewer mistakes. Active learners, meanwhile, tend to test hypotheses quickly and make hasty decisions based on partial information or without thorough analysis; thus, they respond faster but are more prone to errors.

In light of the above, to choose a stimulus mode that would be accurate and efficient, the following questions needed to be considered:

(1) How could we effectively stimulate individual differences in the subjects’ learning styles in the processing dimension?
(2) How could we ensure that the designed stimulus mode would generate as few invalid signals as possible (e.g., from insufficient time for subjects’ information processing or bodily movements that would interfere with the quality of the internal signals)?

#### 2.2.2. Confirming the Stimulus Source

Based on the above, we chose Raven’s Advanced Progressive Matrices (RAPM), developed by British psychologist J.C. Raven in 1992, as the stimulus source for the experiment. 

RAPM could effectively stimulate subjects’ learning-style differences in the processing dimension for the following two reasons.

**Figure 4:**
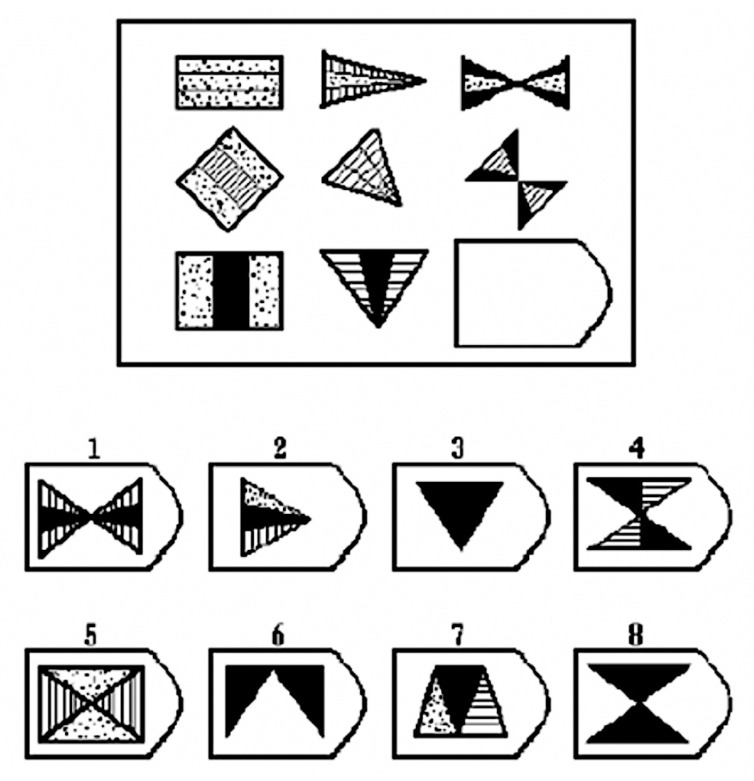
Schematic diagram of an RAPM test question.

(1) RAPM asks subjects to think logically based on the rules associated with the symbols in the matrix diagram. They must fill in vacant positions using the appropriate options. Figure 4 shows the schematic diagram for RAPM test questions. RAPM is often used to assess thinking ability, observational ability, and the ability to use information to solve problems. Using RAPM as a stimulus can prompt subjects to undertake logical thinking that will stimulate brain processing.

(2) Easy questions will reduce the length of cognitive processing, but too difficult ones will generate fatigue and cognitive load, which will affect the quality of the signals. The overall difficulty level of the RAPM is moderate, which can ensure good signal quality. Besides, for younger subjects, the Raven’s Standard Progressive Matrices (RSPM) can adaptively be used instead of the RAPM.

Using RAPM as the stimulus source can reduce the generation of invalid signals in the following two ways.

(1) The RAPM test is largely nontextual. Thus, since subjects do not need to read (test questions, for example), it will reduce the amount of noncognitive processing, which will ensure to the greatest extent that the stimulated signals reflect the brain’s thinking processes.
(2) The RAPM items are presented in the form of multiple-choice questions. Subjects can click the corresponding option to complete their response, which minimizes unnecessary body movement. This can reduce the influence of body movement and other signals on the data.

### 2.3. Collecting the EEG Data

#### 2.3.1. Data-Collection Apparatus

**Figure 5:**
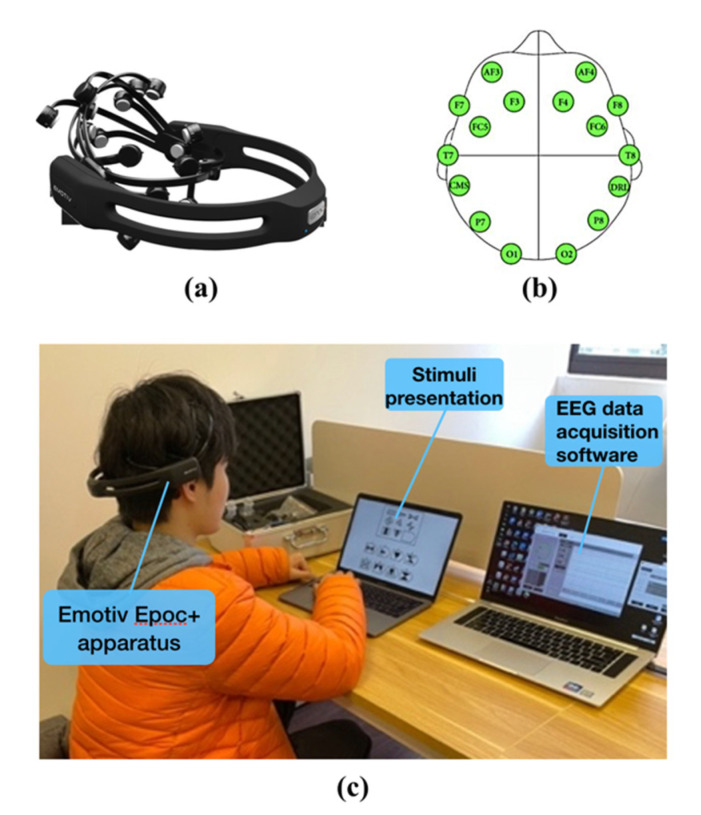
(**a**) Emotiv Epoc+, (**b**) electrodes of Emotiv Epoc+, and (**c**) experimental environment.

A wireless EEG instrument with noninvasive electrodes called Emotiv Epoc+ (Emotiv Systems, San Francisco, USA) was selected for EEG data collection (Figure 5a). It has 14 data-collection channels with a sampling rate of 128 Hz. The electrodes were placed according to the International 10–20 system, as shown in Figure 5b. Although the sensitivity of the Emotiv Epoc+ is not as good as that of a medical-level device, it is relatively affordable and portable, and it has been successfully used in research on cognitive load [52], figure understanding [53], and silent reading [54], among others.

#### 2.3.2. Data-Collection Environment

Data collection was undertaken in a quiet, comfortable laboratory (Figure 5c). The subject wore a brain-computer device. One computer presented the stimulus source while another recorded the raw EEG signals. 

To accurately record the subjects’ answering time and results, E-Prime 2.0 was used to present the stimulus. The timestamp generated by E-Prime 2.0 can be synchronized with the timing of the EEG signal so that the EEG data can be accurately segmented according to subjects’ thinking processes.

#### 2.3.3. Data-Collection Process

**Figure 6:**
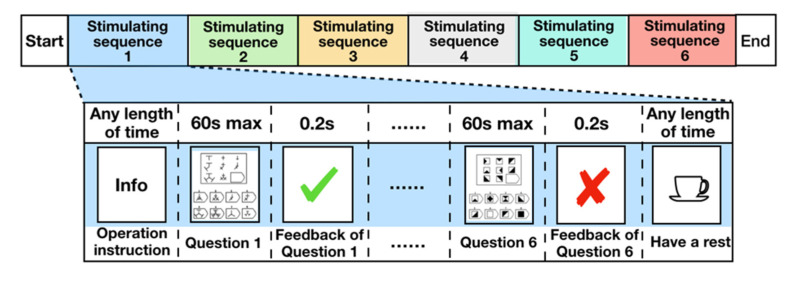
Sequence diagram of one stimulus sequence.

Before starting the data collection, we introduced the experimental procedure and basic operational steps to the subjects and asked them to try to avoid head or body movement. Thirty-six test questions from the stimulus source were equally divided into six sequences; thus, one stimulus sequence consisted of six questions. Figure 6 shows the sequence diagram of a single stimulus sequence. Each stimulus sequence began at the preparation interface. To fully stimulate the subjects’ information-processing signals, the upper limit time of each question was set to 60 seconds; the subjects would then receive feedback on their answer. To prevent cognitive overload and fatigue, there was a rest period after each set of six questions. After the subjects had rested, they pressed a key to resume. The test process ended after all questions were completed.

### 2.4. Preprocessing the EEG Data 

#### 2.4.1. Extraction of Effective EEG Data Segments

**Figure 7:**
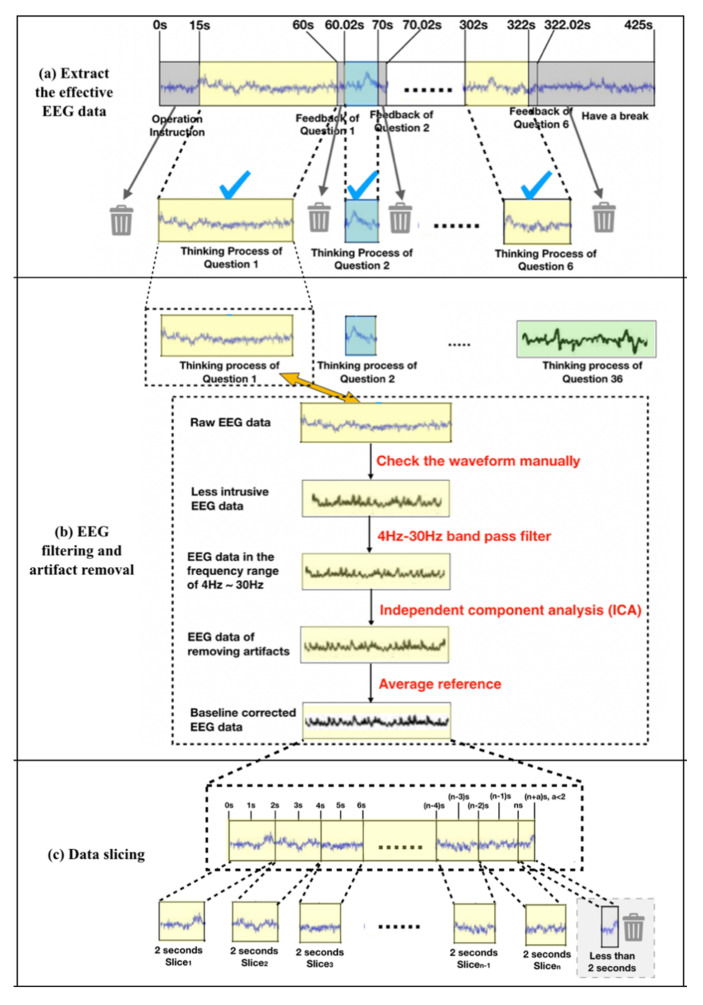
Schematic diagram of data preprocessing.

The raw EEG data contained the subjects’ continuous and total EEG data from the beginning to the end of the experiment. The data included rest time, time taken to answer questions, and other stretches of time that lacked effective cognitive information processing. Therefore, it was necessary to extract the data reflecting effective cognitive information processing. As shown in Figure 7a, the raw continuous signal was sliced according to the time stamp recorded by E-Prime in the background, and EEG signals corresponding to effective information processing for each question were obtained. 

A total of 504 pieces of experimental data were obtained (14 subjects, 36 questions each), with durations ranging from 5 to 60 s.

#### 2.4.2. EEG Filtering and Artifact Removal

Since the EEG data contained a large amount of artifact data (e.g., electrooculogram (EOG), electromyogram (EMG), and power frequency interference), it was necessary to filter that data out. Figure 7b shows the data-filtering process. In the experiment, the raw EEG data were preprocessed through the following steps. First, data segments with obvious interference were removed manually by checking the waveform. Then, since the effective range of the human brain’s EEG signal is 0–30 Hz, and the delta band (0–4 Hz) of the EEG signal only appears during deep sleep, effective EEG data in the range of 4 Hz–30 Hz were obtained using EEGLAB to perform band-pass filtering. In addition, during the experiment, blinking, hand movement, and other actions interfered with the EEG signal. Thus, independent component analysis (ICA) was used to remove artifacts such as EOG and EMG. Finally, considering that people’s brainwave benchmarks are not the same, some brainwave signals were stronger while others were weaker. Thus, we used the first two seconds of each piece of EEG data as the baseline and used the average reference method to normalize the EEG data. Finally, clean EEG signals were obtained.

#### 2.4.3. Data Slicing

To solve the problem of variable-length data input and to increase the number of training samples, a slicing method was used to slice the EEG data. Figure 7c shows the slicing operation. EEG data corresponding to each question were divided into several data slices (from Slice_1 to Slice_n) with a duration of 2 s by the time-sliding window. There was no overlap between the two data slices, and data under 2 s were ignored. After data slicing, a total of 8358 EEG data slices with a 2 s duration were generated.

#### 2.4.4. Labeling EEG Data to Be Trained

The processed data slices were labeled with the actual learning-style labels obtained from the ILS (Section 2.1). All slices were randomly ordered and used as training samples for the recognition model.

### 2.5. Constructing the Recognition Model 

**Figure 8:**
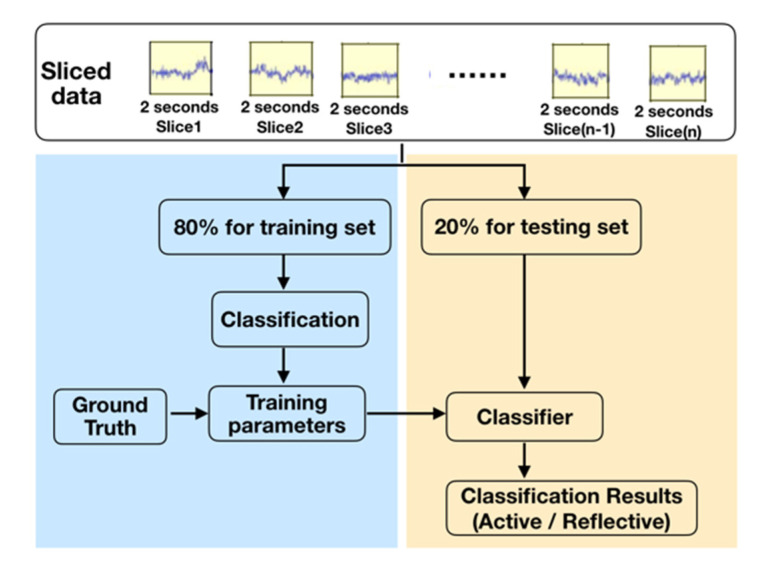
Model construction process.

Model construction mainly included two parts: training and recognition. Figure 8 shows the process. Model construction was performed using Python programming under the Pytorch framework. The system specifications were Intel(R) Core(TM) i5-9300H CPU @ 2.40 GHz processor, 32 GB memory, NVIDIA GeForce RTX 2080Ti graphics card, and 64-bit Windows 10 OS.

#### 2.5.1. Training Process

Before model training, 80% of the 8358 labeled data was randomly sampled as the training set and 20% as the testing set with no overlap between the two. Support vector machines (SVMs) and backpropagation neural networks (BPs) are widely used in classification scenarios; thus, we trained using SVM and BP models. In the training process, feature extraction and parameter updating were performed on the preprocessed experimental data. 

In addition, considering that EEG signals contain high-dimensional time, space, and frequency features, we used a convolutional neural network (CNN) model to construct a one-dimensional multiscale spatiotemporal convolutional neural network model (1-DCNN) to optimize the accuracy of existing EEG recognition models. The specific measures were as follows: first, we used a one-dimensional spatiotemporal convolution kernel instead of the traditional two-dimensional kernel, which can extract the spatiotemporal features of EEGs between channels and reduce the model training parameters. Second, we built a parallel multiscale convolution module, which can effectively obtain more abundant EEG features. Third, we replaced the fully connected layer with global average pooling, which can effectively increase training speed and minimize the effect of overfitting.

#### 2.5.2. Recognition Process

**Figure 9:**
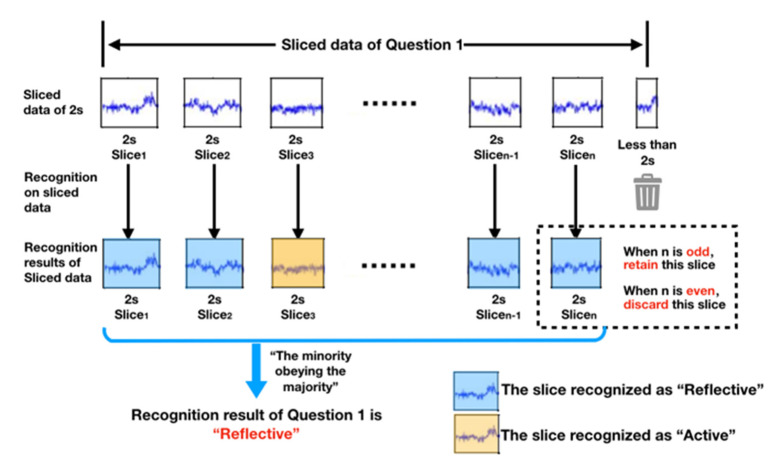
Principle of the recognition mechanism.

In the recognition process, the trained parameters were directly used to construct a recognition model for learning-style classification. Then, the testing set was used to evaluate the performance of the model. In recognition, according to the classification results of the model for the slices, the strategy of “the minority obeys the majority” was used; that is, the category that appeared the most was used as the final classification label for the whole thinking process (Figure 9). To ensure there was only one classification result for a topic and to keep active and reflective styles from occurring simultaneously, it was necessary to ensure that the number of slices of the question was an odd number. Thus, we deleted the last slice when the data slices were even.

## 3. Experimental Results and Analysis

### 3.1. Verifying the Experimental Design

#### 3.1.1. Data Visualization Analysis of Subjects’ Experimental Results

**Figure 10:**
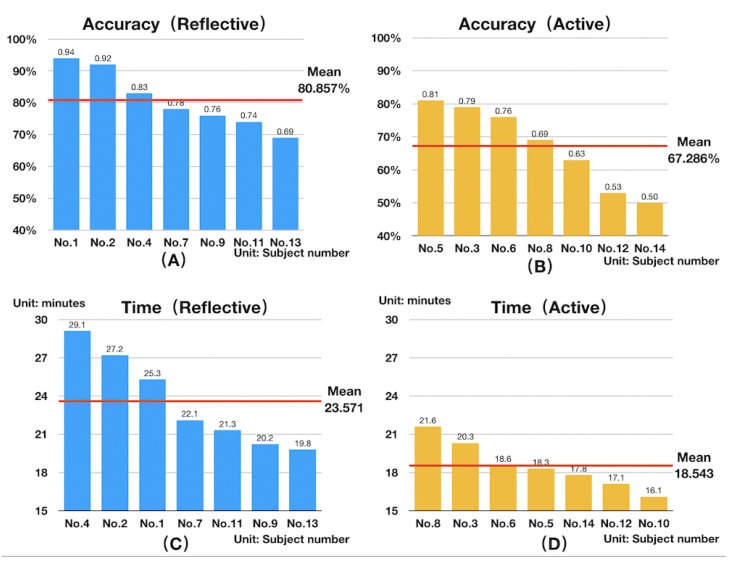
(**A**) Answer accuracy by reflective subjects, (**B**) answer accuracy by active subjects, (**C**) time used by reflective subjects, and (**D**) time used by active subjects.

We analyzed the subjects’ actual learning-style labels and their behavior differences to verify the effectiveness of the experimental design. The results obtained by analyzing the subjects’ answer times and correct rates in the experiment are visualized in Figure 10. 

Figure 10A,B show the answer accuracy of reflective and active learners, respectively. The abscissa is the subject’s number, and the ordinate is the subject’s answer accuracy. Figure 10A,B show that the reflective learners’ average accuracy rate was 80.857% (standard deviation: 8.626%) while that of active learners was 67.286% (standard deviation: 11.498%). The overall accuracy rate of reflective subjects was higher than that of active subjects, which is consistent with the differences in the accuracy rates of learners in the Felder–Silverman processing dimension.

Figure 10C,D show the total answer times of reflective and active learners, respectively. The abscissa is the subject number, and the ordinate is the answer time of each subject. The average time taken by reflective learners was 23.571 min (standard deviation: 3.373 min) while that of active learners was 18.543 min (standard deviation: 1.736 min). Thus, reflective subjects took more time than active subjects, which aligns with the differences in the answer times of learners in the Felder–Silverman processing dimension. 

The analysis of subjects’ learning behavior during the experiment shows that active learners spent less time and had lower accuracy in solving problems while reflective learners spent more time and had higher accuracy. The experimental process was therefore able to distinguish the behavior differences between the two learning styles in the processing dimension, demonstrating the rationality of the experimental design.

#### 3.1.2. Statistical Analysis

We used the Chi-square test [51] to assess if there exists an association between the answer results (correct/wrong) and the processing dimension of the learning style (reflective/active). On the contrary, we used the Mann–Whitney U test [55] to compare the answer time lengths (quantitative variable) between the two types of learning styles in the processing dimension.

##### Verify Significant Differences in Answer Results

First, we compared the answer results of the two groups of learners with different learning styles in the processing dimension (active/reflective) by Chi-square test. Regarding the question, “Do the different learning styles in the processing dimension (active/reflective) affect the answer results (correct/wrong)?” we propose the hypotheses shown in the Equations (1) and (2), where *P_a_^c^* is the proportion of correct answer results in the active learners’ group, whereas *P_r_^c^* is the proportion of correct answer results in the reflective learners:

*H0*: *P_a_^c^* = *P_r_^c^* (1)

*H1*: *P_a_^c^* ≠ *P_r_^c^* (2)

Table 3 shows the contingency table results for the answer results of the two types of learning styles. There are 504 single-answer results of the two groups of learners (reflective/active): 204 of them are answered correctly by reflective learners and 169 of them are answered correctly by active learners. 

The result of the Chi-square test shows that χ^2^ statistic = 11.9236(degree of freedom = 1), *p* = 0.0006; thus, hypothesis *H0* in Formula (1) is rejected, and *H1* in Formula (2) is accepted. This indicates that the learning styles in the processing dimension (active/reflective) have a significant impact on the answer results (correct/wrong); that is, it was verified that there was a significant difference in answer results between the two groups of subjects with different learning styles.

##### Verify Significant Differences in Answer Time

Similarly, we compared answer times between the two groups with different learning styles by Mann–Whitney U test. Regarding the question, “Is the length of answer time of active learners significantly different from the one of reflective learners?” we propose the hypotheses shown in Formulas (3) and (4), where *M_a_^c^* and *M_r_^c^* are the median values of subjects’ answer times:

*H0*: *M_a_^c^* = *M_r_^c^* (3)

*H1*: *M_a_^c^* ≠ *M_r_^c^* (4)

The result of the Mann–Whitney U test shows that U statistic = 5.0000, *p* = 0.0127; thus, hypothesis *H0* in Formula (3) is rejected, and *H1* in Formula (4) is accepted. This indicates that there were significant differences in the answer time of the two groups of subjects with different learning styles.

##### Analysis of Statistical Conclusion

It has been proven that there were statistically significant differences in the answer accuracy and answer time of subjects with different learning styles. This further verifies the rationality of the experimental design in this study—namely, its ability to evoke the internal-state differences of different learners in the processing dimension.

### 3.2. Effectiveness of EEG-Based Learning-Style Recognition

**Figure 11:**
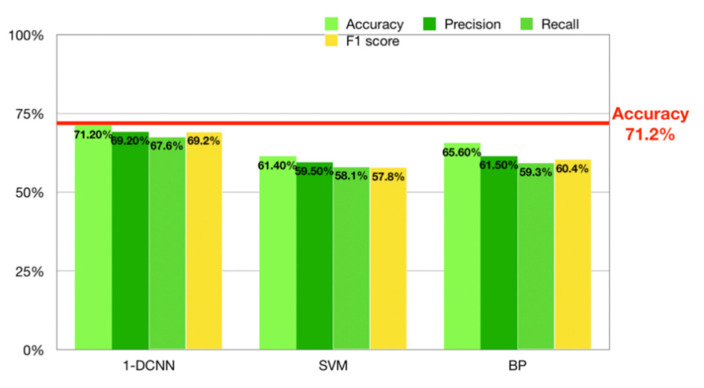
Comparison of the recognition accuracy, precision, recall, and F1 score of different models.

We used the processed EEG signals as the data source for the recognition model and analyzed its accuracy using the test set to verify the effectiveness of EEG-based learning-style recognition. In this study, two commonly used recognition models (SVM and BP) and one optimized recognition model based on CNN (1-DCNN) were used to perform recognition using the testing set. Figure 11 shows the results of different models on accuracy, precision, recall, and F1 score. It can be seen that our optimized 1-DCNN model achieved a recognition accuracy of 71.2%, a precision of 69.2%, a recall of 67.6%, and an F1 score of 69.2%, demonstrating its ability to recognize learning style. Therefore, it was verified that learning-style recognition based on EEG signals is reasonable. Figure 11 also shows that our optimized 1-DCNN recognition model could improve the accuracy of EEG-based learning-style recognition models. 

To further verify that EEG features can be effectively applied to learning-style recognition, we compared the recognition result of our experiment with the results of other Felder–Silverman learning-style dimensions with regard to data sources and recognition precision. 

Table 4 shows that, compared to other mature learning-style recognition methods based on online interactive behavior logs, the precision of our proposed EEG-based recognition method is promising. The recognition precision of our approach has great potential for improvement by, for example, using higher-precision data-collection equipment, increasing the number of subjects, and further optimizing the classifier structure to obtain better recognition precision. In this way, a new field of EEG-based learning-style recognition can potentially be developed.

## 4. Discussion and Conclusions

Aiming to overcome the problems of existing learning-style recognition mechanisms, this study developed a novel learning-style recognition mechanism based on EEG. The mechanism involved (1) labeling learners’ actual learning styles, (2) designing stimuli to evoke learners’ internal state signals, (3) designing the data-collection method, (4) designing the preprocessing procedure, and (5) constructing the recognition model. 

This study’s contributions are as follows:

(1) We designed and verified an experimental method that effectively stimulated internal state differences in the subjects’ different learning styles in the processing dimension. Based on Felder–Silverman’s processing-dimension theory, we conducted an experiment to stimulate subjects’ state differences in the processing dimension. The validity of the processing-dimension state differences stimulated by the experiment was then verified through a statistical analysis of the subjects’ behavioral states.
(2) We confirmed the validity of learning-style recognition based on EEG signals. We designed an appropriate experimental acquisition environment, collected EEG signals from the subjects’ processing dimension, processed the collected EEG data, and constructed a 1-DCNN model for recognition. The recognition result was 71.2%, showing that an EEG-based learning-style recognition algorithm has promising classification ability. This also confirmed that the 1-DCNN recognition algorithm could improve the accuracy of the EEG-based learning-style recognition model. In addition, we compared the accuracy of the proposed method with that of other mature recognition methods and further verified the effectiveness and potential of EEG-based learning-style recognition.

In summary, our proposed EEG-based learning-style recognition mechanism has important significance for learning-style recognition. In the future, we will further optimize recognition accuracy in terms of four aspects: the quality and quantity of data sources, the method for preprocessing EEG data, and the structure of the recognition model. We will also continue to investigate the use of EEG features to recognize other learning-style dimensions.

## Figures and Tables

**Table 2:** Internal activity state of the human brain corresponding to the frequency band of EEG signal.

EEG Signal Band	Frequency	Meaning
Delta	0.5 Hz to 4 Hz	Deep sleep [44]
Theta	4 Hz to 7 Hz	Drowsiness or mediation [44], working memory and processing [45]
Alpha	8 Hz to 12 Hz	Sensory suppression mechanism during selective attention [46], awakening [44], inhibition of irrelevant stimuli [45]
Beta	13 Hz to 30 Hz	Active thinking and attention, outside world, and problems solving [47]
Gamma	Above 30 Hz	Consciousness [48], cognitive control during detecting emotional expressions [49]

**Table 3:** Contingency table for the answer results and the two types of learning styles in the processing dimension (reflective/active).

Answer Results\Processing Dimension	Reflective Learners	Active Learners
Correct	204	169
Wrong	48	83

**Table 4:** Comparison of the proposed mechanism and other recognition mechanisms in terms of data sources and recognition precision.

Method	Data Source	Precision
Proposed	EEG features	69.2%
Quang and Florea, 2012 [56]	Online interactive behavior log	72.7%
Karagiannis and Satratzemi, 2017 [57]	Online interactive behavior log	70.0%
Liyanage et al., 2014 [58]	Online interactive behavior log	65.0%
Kappel and Graf, 2007 [59]	Online interactive behavior log	62.5%
Ömer et al., 2010 [50]	Online interactive behavior log	79.6%
Bernard et al., 2017 [60]	Online interactive behavior log	81.9%

## Data Availability

The original contributions presented in the study are included in the article; further inquiries can be directed to the corresponding authors.
